# Total Micronutrient Intake and Epigenetic Aging in the Women’s Health Initiative

**DOI:** 10.1093/geroni/igaf122.3333

**Published:** 2025-12-31

**Authors:** Lindsay Reynolds, Alexandra Cowan-Pyle, Regan Bailey, Diane Mitchell, Eric Whitsel, Mara Vitolins, Janet Tooze

**Affiliations:** Wake Forest University School of Medicine, Winston-Salem, North Carolina, United States; Texas A&M University, College Station, Texas, United States; Texas A&M University, College Station, Texas, United States; Texas A&M University, College Station, Texas, United States; The University of North Carolina at Chapel Hill Gillings School of Global Public Health, Chapel Hill, North Carolina, United States; Wake Forest University School of Medicine, Winston-Salem, North Carolina, United States; Wake Forest University School of Medicine, Winston-Salem, North Carolina, United States

## Abstract

Use of dietary supplements (DS) is common among older adult women and represents an important source of micronutrient intake. To understand whether recommended micronutrient intake is related to slower biological aging, we estimated associations between DS use, total micronutrient intake (from diet and DS use), and an epigenetic biomarker of pace of aging (DunedinPACE). We conducted a cross-sectional study of 4,582 postmenopausal women aged 50–79 years at the Women’s Health Initiative (WHI) baseline visit (1993-1998), leveraging food frequency questionnaire, DS use, and leukocyte DNA methylation (DNAm) data. We calculated micronutrient intake using the Total Nutrient Index (TNI, range: 0-100), higher values of which indicate greater adherence to recommended Dietary Reference Intakes for six under-consumed micronutrients (calcium; magnesium; vitamins A, C, D, and E from diet and DS) among the U.S. population. We used linear mixed models adjusted for age, self-identified race and ethnicity, education, smoking, physical activity, ancillary study DNAm data source, and leukocyte proportions to estimate DS use- and TNI-DunedinPACE associations. DS use was common among participants (60%) and associated with a slower DunedinPACE (Beta±SE: -0.100 ±0.026, p = 1.5E-4). A higher TNI score also was associated with a slower DunedinPACE (Beta±SE: -0.040 ±0.013, p = 0.0022). The TNI micronutrient component most strongly associated with DunedinPACE was vitamin C (Beta±SE: -0.0025 ±0.0007, p = 1.19E-04). Other TNI components inversely associated with DunedinPACE were calcium (p = 5.9E-4), magnesium (p = 0.021), and vitamin E (p = 0.012). These findings support that DS use and recommended micronutrient intakes are associated with epigenetic aging and may influence the rate of aging.

